# Affection of facial artifacts caused by micro-expressions on electroencephalography signals

**DOI:** 10.3389/fnins.2022.1048199

**Published:** 2022-11-24

**Authors:** Xiaomei Zeng, Xingcong Zhao, Shiyuan Wang, Jian Qin, Jialan Xie, Xinyue Zhong, Jiejia Chen, Guangyuan Liu

**Affiliations:** ^1^School of Electronics and Information Engineering, Southwest University, Chongqing, China; ^2^Institute of Affective Computing and Information Processing, Southwest University, Chongqing, China; ^3^Chongqing Key Laboratory of Nonlinear Circuits and Intelligent Information Processing, Southwest University, Chongqing, China

**Keywords:** micro-expression, electroencephalography (EEG), artifacts, multiple linear regression analysis, Granger causality test

## Abstract

Macro-expressions are widely used in emotion recognition based on electroencephalography (EEG) because of their use as an intuitive external expression. Similarly, micro-expressions, as suppressed and brief emotional expressions, can also reflect a person’s genuine emotional state. Therefore, researchers have started to focus on emotion recognition studies based on micro-expressions and EEG. However, compared to the effect of artifacts generated by macro-expressions on the EEG signal, it is not clear how artifacts generated by micro-expressions affect EEG signals. In this study, we investigated the effects of facial muscle activity caused by micro-expressions in positive emotions on EEG signals. We recorded the participants’ facial expression images and EEG signals while they watched positive emotion-inducing videos. We then divided the 13 facial regions and extracted the main directional mean optical flow features as facial micro-expression image features, and the power spectral densities of theta, alpha, beta, and gamma frequency bands as EEG features. Multiple linear regression and Granger causality test analyses were used to determine the extent of the effect of facial muscle activity artifacts on EEG signals. The results showed that the average percentage of EEG signals affected by muscle artifacts caused by micro-expressions was 11.5%, with the frontal and temporal regions being significantly affected. After removing the artifacts from the EEG signal, the average percentage of the affected EEG signal dropped to 3.7%. To the best of our knowledge, this is the first study to investigate the affection of facial artifacts caused by micro-expressions on EEG signals.

## Introduction

Emotion is a vital element in the daily lives of human beings, and personal emotional states may become apparent through subjective experiences and internal/external expressions (LeDoux and Hofmann, 2018), which have an important impact on various aspects of human social interaction (Huang et al., 2017), behavioral regulation (Dzedzickis et al., 2020), and mental health (Inwood and Ferrari, 2018). Therefore, research on emotion recognition has important theoretical and practical implications. Previous research has primarily focused on the analysis of human facial expressions (Abdat et al., 2011; Li et al., 2019a), behaviors, and physiological signals (Zheng et al., 2017; Shu et al., 2018; Egger et al., 2019), such as facial images, electroencephalography (EEG), galvanic skin response (GSR), and heart rate (HR), which can be used to effectively evaluate personal emotional states. Among them, EEG signals are used extensively in emotion recognition studies because of their objective realism and high temporal resolution. As an external expression of emotion, facial macro-expressions are used as one of the most reliable emotion indicators (Tan et al., 2021). Therefore, researches on emotion recognition based on macro-expressions and EEG signals have attracted significant attention (Matlovic et al., 2016; Huang et al., 2019).

Micro-expressions, as suppressed emotional expressions, are typically very brief, involuntary facial expressions that occur when a person is trying to hide, fake, or suppress their genuine emotions, and last around 1/25–1/2 s (Ekman and Friesen, 1969; Ekman, 2009). Studies have shown that micro-expressions contain a significant and effective quantity of information about the true emotions, which is useful in practical applications such as lie detection, justice, and national security (Frank et al., 2009; Ten Brinke et al., 2012; Yan et al., 2013). With the development of computer vision technology, research on image-based micro-expression recognition has made significant progress. For example, Huang et al. (2016) proposed spatio-temporal completely local quantized patterns (STCLQP) and Xia et al. proposed a recurrent convolutional network (RCN) (Xia et al., 2020b) as well as a spatio-temporal recurrent convolutional network (STRCN) (Xia et al., 2020a), which achieved favorable recognition performance for spontaneous micro-expression recognition. Research on emotion recognition based on micro-expressions and EEG signals is currently underway (Kim et al., 2022; Saffaryazdi et al., 2022; Wang et al., 2022). However, EEG signals are contaminated by facial muscle activity, which is termed “muscle artifacts” (Barlow, 1986; Fatourechi et al., 2007; Whitham et al., 2007; McMenamin et al., 2011). These artifacts can cover the power spectrum of EEG signals and affect their ability to detect the functional state of the brain.

Goncharova et al. (2003) investigated the spectral and topographical characteristics of the facial artifacts caused by contractions of the frontalis and temporalis muscles over the entire scalp. They found that facial artifacts affect the EEG signal, where contractions of the temporal and frontal muscles significantly affect the temporal and frontal regions (Whitham et al., 2007; Yong et al., 2008). Similarly, Soleymani et al. (2015) supported this view in their study, using EEG signals and facial macro-expressions for continuous emotion recognition and analyzing the correlation between facial expressions and EEG signals. They noted that facial artifacts significantly affect the EEG signal, mainly affecting electrodes on the frontal, parietal, and occipital regions, and that such artifacts are more pronounced in the higher frequencies (beta and gamma bands), with an average degree of effect percentage of up to 54%. Compared with macro-expressions, micro-expressions are weak and short-term (Porter and Ten Brinke, 2008; Ekman, 2009; Shreve et al., 2009). Therefore, the artifacts caused by micro-expressions may affect the EEG signal to a lesser extent. To explore the degree and mode of effect on the EEG signal caused by micro-expression artifacts, we proposed a study to analyze the effect of artifacts caused by micro-expressions on EEG signals.

In this study, we analyzed the correlation between facial micro-expressions and EEG signals, aiming to address three questions: (1) whether facial muscle artifacts caused by micro-expressions affect EEG signals; (2) in case of an effect, how much of the facial artifact affects the EEG signals and what are the main brain regions that are affected; (3) whether the effects on EEG signals can be eliminated after the artifacts are removed. In addition, we validated the method by analyzing the correlation between macro-expressions and EEG signals. The remainder of this paper is structured as follows: In Section “Data collection,” the data collection process is described. Section “Materials and methods” details the analysis of the correlation between EEG signals and facial micro/macro-expressions and their relationship. The experimental results are presented in Section “Results.” Section “Discussion” provides the discussion of this study. Finally, conclusions are presented in Section “Conclusion.”

## Data collection

### Participants

A total of 78 right-handed healthy participants were recruited for this experiment. None of them had a history of neurological or psychiatric diagnosis. Of these 78 participants, 10 were excluded owing to ineligibility, and the remaining 68 (age range: 17–22 years, 23 males, 45 females) had valid data. Written informed consent was obtained from all participants after all experimental procedures were approved by the Ethics Committee of Southwest University.

### Material

Emotional elicitation can be achieved using external stimuli and internal responses. The stimulation methods commonly used by researchers aim to induce different emotions in participants through external stimuli, such as pictures (Mehmood and Lee, 2016), music (Bhatti et al., 2016), and videos (Li et al., 2019b). In recent years, an increasing number of emotion recognition studies have started using a combination of audio-visual stimuli to induce emotions, and widely used micro-expression datasets currently use videos as emotion-evoking materials (Davison et al., 2016; Qu et al., 2017). Therefore, in our experiment, we used video clips with high emotional valence as stimulus materials to induce emotions in the participants.

Previous studies have shown that happiness is more easily induced than sadness (Coan and Allen, 2007). Therefore, this study aimed to induce positive emotions. Considering cultural differences, we downloaded seven video clips from different regions of the Internet for emotion expression inhibition, which were considered highly positive in terms of valence. The length of the selected videos was approximately 2 min to avoid visual fatigue, and each video primarily induced a single target emotion. Before the videos were presented to the participants, we carefully assessed the valence of these videos and rated the intensity from 0 to 7, with 0 being the weakest and 7 being the strongest.

### Procedure

The participants were requested to sit in front of a computer and a webcam. The researcher adjusted the participants’ chairs to keep their faces facing the webcam and instructed the participants to keep their head posture as still as possible throughout the experiment. Before the start of the experiment, the participants were requested to watch a video to familiarize them with the entire experimental process, and no EEG data were collected. After ensuring that both the experimental equipment and the EEG channel signals were intact, a formal experiment was conducted.

**FIGURE 1:**
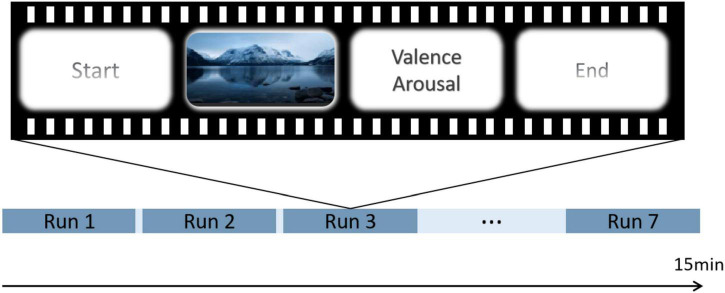
Example of the experimental process operation.

Throughout the experiment, each run contained one video and the participants completed seven video views. At the beginning of each video, a gaze cross appeared in the center of the screen, and the participants responded accordingly to the instructions that appeared on the screen. After each video, the participants were requested to rate their perceived valence and arousal at that moment. First, we played a neutral video to ensure that participants were in a calm state. During the emotion-control task phase, participants were requested to focus on the video. Additionally, they were required to suppress their facial expressions while watching the video to avoid revealing their true emotions. The experimental procedure is shown in Figure 1.

We added stress to the classical micro-expression paradigm, such that participants are in a situation of high-intensity emotional arousal and strong motivation to suppress their facial expressions. This was done because micro-expressions appear in high-risk situations (Goh et al., 2020). Participants and supervisors participated in the experiment simultaneously, with the participants separated from the supervisors using a curtain to allow them to concentrate on completing the experiment. The supervisor observed the participants’ facial micro-expressions on a monitor. If the supervisor found that the participant did not suppress his or her facial expressions while watching the video, a part of the participant’s reward was deducted. This allowed the participants to hide their true inner emotions better to ensure the reliability of the micro-expressions.

### Signal acquisition

The participants’ facial images were acquired at 100 fps using a high-speed camera to record their facial expressions. EEG signals were recorded using a Biosemi Active system (ActiveTwo Acquisition 125 system, Netherlands) with 128 Ag/AgCl electrodes. The sampling rate was 1,024 Hz. In addition, to ensure that the EEG signal and facial images were acquired accurately and synchronously, we used the same trigger to generate timestamps on both the camera recording and the Biosemi Active system.

## Materials and methods

### Dataset construction

Dataset construction is an important step in the data analysis phase. In general, facial expression dynamics comprises of three main phases: onset, peak, and offset. These phases appear in any expression, whether it is macro or micro. The essential difference between micro-expressions is the duration of the expression rather than the intensity. Therefore, in this study, partial or complete facial expressions of ≤500 ms were considered micro-expressions, whereas facial expressions of >500 ms were classified as macro-expressions.

To detect the micro-expressions of participants under time-locked stimuli, we first invited two trained coders to calculate the time points for all facial expressions (both micro- and macro-expressions) for all participants. This step was conducted to detect the time points of participants’ facial responses while watching the video. The coders determined the approximate time points of the onset, peak, and offset frames by playing the recordings frame-by-frame. When the coders did not agree on the onset, peak, and offset frames of the expression, the average of the frame values was used.

Subsequently, 2 s segments of the participants’ EEG signals associated with facial expressions were intercepted using the moment of occurrence of the peak of all facial expressions as the midpoint, and the global field power (GFP) (Khanna et al., 2015) of the intercepted 2 s was calculated. The GFP is the root of the mean of the squared potential differences at all *K* channels [i.e., _*V_i_ (t)*_] from the mean of instantaneous potentials across channels [i.e., _*V_mean_ (t)*_]. It is the transient electric field strength of the brain and is often used to describe rapid changes in brain activity.


(1) $$G\,F\,P=\sqrt{\left(\sum_{i}^{k}{\left(V_{i}\,\left(t\right)-V_{m\,e\,a\,n}\,\left(t\right)\right)}^{2}\right)/K}.$$

Therefore, we selected the moment of the maximum peak as the midpoint because the peak of the GFP curve is the maximum field strength. Ultimately, we selected 1 s data with the largest peak (calculated by GFP) as the sample.

Finally, we used statistics to determine the point in time when most participants presented facial expressions (both micro- and macro-expressions). This constituted the dataset used in this study, with 806 micro-expression and 393 macro-expression samples.

### Electroencephalography signal

#### Pre-processing

Electroencephalography signals are weak because they are susceptible to interference from other noise during the acquisition process. Pre-processing of EEG signals refers to the removal of artifacts from the collected EEG signals. In the EEG signal pre-processing stage of this study, we used a finite impulse response (FIR) band-pass filter to filter (0.5–60 Hz), notch filter to remove the 50 Hz power frequency interference and reject bad electrodes. For a better approximation of the ideal zero reference, the EEG signals should be referenced to the Reference Electrode Standardization Technique (REST) using the REST software (Dong et al., 2017). All the aforementioned steps were performed using the eeglab toolbox in MATLAB (Brunner et al., 2013). In addition, we removed artifacts, such as ECG, electrooculography (EOG), and electromyography (EMG) from the EEG signal, which was performed to subsequently make a better comparison of the effects of the removal of artifacts on the EEG signal.

#### Feature extraction

Studies have shown that the five frequency bands of EEG, including the delta, theta, alpha, beta, and gamma bands, are closely related to various physiological and emotional states of human beings (Davidson, 2003; Wagh and Vasanth, 2019). Therefore, when extracting EEG frequency-domain features, many researchers first map EEG signals to these five frequency bands and then extract the frequency-domain features corresponding to each band separately (Jenke et al., 2014; Yin et al., 2017). In this study, 128 electrodes were used for EEG feature extraction. The power spectral densities (PSDs) were extracted from theta (4 Hz < *f* < 8 Hz), alpha (8 Hz < *f* < 12 Hz), beta (12 Hz < *f* < 30 Hz), and gamma (30 Hz < *f*) bands as features, and the PSD was extracted from a 1 s time window with 20% overlap. The total number of EEG features from 128 electrodes and 4 frequency bands was 128 × 4 = 512 features.

### Analysis of facial micro-expressions

**FIGURE 2:**
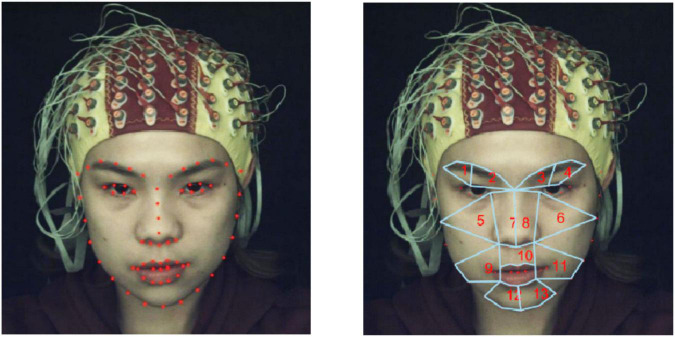
**(Left)** Detection of 68 feature points in facial regions using Dlib. **(Right)** Partitioning of 13 ROIs.

First, we used OpenCV and Python to implement a face detector (Bradski and Kaehler, 2000). The Dlib library uses Dlib’s pretrained facial landmark point detector to detect and locate 68 facial landmark points (Mohanty et al., 2019). The detection results for the 68 facial landmarks are shown in the left of Figure 2.

After 68 facial landmark points were detected, we divided the facial regions into 13 regions of interests (ROIs) based on the coordinates of the facial landmark points and facial action coding system (FACS), as shown in the right of Figure 2. Note that the division of ROIs should not be significantly compact to avoid introducing redundant and unwanted information. Similarly, the division of ROIs should not be significantly sparse to avoid losing potentially useful information. According to the statistics of Liong et al.’s (2015) on the occurrence frequency of face regions based on action units (AUs), it was found that the eyebrows and mouth were the regions that appeared most frequently when micro-expressions occurred; therefore, we divided the mouth and eyebrow regions more carefully. Regions such as cheeks were divided more sparsely. The 13 ROIs and corresponding AUs divided in this study are listed in Table 1.

**TABLE 1:** Thirteen ROIs and corresponding action unit (AU) information.

ROIs	AUs
ROI1	AU2, AU5
ROI2	AU1, AU4
ROI3	AU1, AU4
ROI4	AU2, AU5
ROI5	AU6, AU13
ROI6	AU6, AU13
ROI7	AU9
ROI8	AU9
ROI9	AU6, AU10, AU12, AU13, AU14, AU15, AU16, AU18
ROI10	AU10
ROI11	AU6, AU10, AU12, AU13, AU14, AU15, AU16, AU18
ROI12	AU16, AU17, AU18, AU20, AU23, AU24, AU26
ROI13	AU16, AU17, AU18, AU20, AU23, AU24, AU26

**FIGURE 3:**
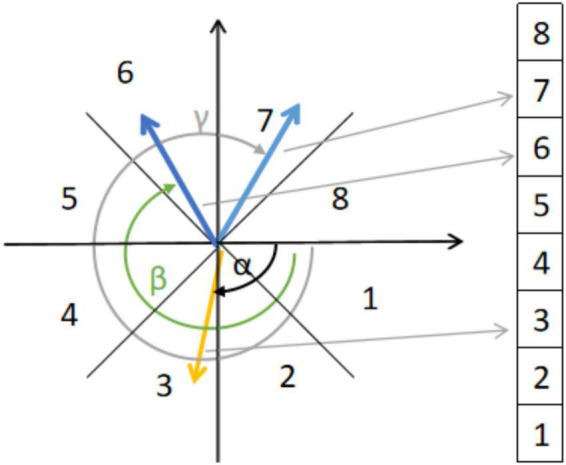
Histogram of oriented optical flow with eight bins.


For a micro-expression video sequence, we then calculated the optical flow between the first frame *f*_1_ and each subsequent frame *f*_*i*_ and denoted it as [u_i_,v_i_]T (Zhao and Xu, 2019). The Euclidean coordinates [u_i_,v_i_]T were converted to polar coordinates (ρ_i_,θ_i_). ρ_*i*_ and θ_*i*_ are the magnitude and direction of the optical flow vector, respectively. A location $p\,\epsilon \,R_{i}^{k}$ is represented as $\varphi_{\text{i}}^{\text{k}}\,\left(\text{p}\right)=\left(\rho_{\text{i}}^{\text{k}}\,\left(\text{p}\right),\theta_{\text{i}}^{\text{k}}\,\left(\text{p}\right)\right)$, where *i* = 1,2,3…,*n*_*f*_ is the frame index, in which *k* = 1,2,3…,13 is the index of the ROIs. The optical flow within each ROI was divided into eight directional intervals based on the optical flow direction, as shown in Figure 3. The optical flow direction histogram (HOOF) was subsequently calculated. Based on the resulting histogram of optical flow directions, we selected the bin with the largest number of optical flow vectors and calculated its mean value as the feature vector using Eq. 2:

(2) $${\bar{\varphi }}_{i}^{k}=\frac{1}{\left|B_{m\,a\,x}\right|}\,\sum_{\varphi_{i}^{k}\,\left(p\right)\,\epsilon \,B_{m\,a\,x}}\varphi_{i}^{k}\,\left(p\right).$$

where *B*_*max*_ is the set of optical flow vectors that fall into the largest number in the bin and |.| is the set base.

${\bar{\varphi }}_{i}^{k}=\left({\bar{\rho }}_{i}^{k},{\bar{\theta }}_{i}^{k}\right)$, ${\bar{\theta }}_{i}^{k}$ is the main direction of the optical flow of $R\,O\,I\,R_{i}^{k}$. Therefore, the feature vector of frame *i* is


(3) $$\sigma_{i}=\left({\bar{\varphi }}_{i}^{1},{\bar{\varphi }}_{i}^{2},\ldots ,{\bar{\varphi }}_{i}^{13}\right).$$

Subsequently, the dimension of the feature vector σ_*i*_ is 13 × 2 = 26, where 13 is the number of ROIs.

After the optical flow feature extraction is completed for each frame, the optical flow feature value for a micro-expression sequence is *U* = (σ_1_,σ_2_,…,σ_*i*_). Among them, *i* is the number of frames of the micro-expression sequence. We first convert all polar coordinates ${\bar{\varphi }}_{i}^{k}$ to Euclidean coordinates $\omega \,\left({\bar{\varphi }}_{i}^{k}\right)=\left(x_{i}^{k},y_{i}^{k}\right)$, $\omega \,\left(\sigma_{i}\right)=\left(\omega \,\left({\bar{\varphi }}_{i}^{1}\right),\omega \,\left({\bar{\varphi }}_{i}^{2}\right),\ldots ,\omega \,\left({\bar{\varphi }}_{i}^{13}\right)\right)$ and subsequently calculate its average value as the main directional mean optical flow (MDMO) feature:


(4) $$\omega \,\left(\bar{\sigma }\right)=\frac{1}{n_{f}}\,\sum_{i=1}^{n_{f}}\omega \,\left(\sigma_{i}\right).$$

Then, the final MDMO features of the micro-expression sequence are


(5) $$\bar{\sigma }=\left[\left({\bar{\rho }}_{1},{\bar{\theta }}_{1}\right),\left({\bar{\rho }}_{2},{\bar{\theta }}_{2}\right),..,\left({\bar{\rho }}_{13},{\bar{\theta }}_{13}\right)\right].$$

### Analysis of facial macro-expressions

Macro-expression feature extraction is similar to micro-expression feature extraction, except for one key difference. The main difference is that because macro-expressions generate more facial movements when they appear, which occur over the whole face, they can cover a large area of the face region. Therefore, during macro-expression feature extraction, we did not divide the facial region into 13 sub-regions. Here, we extracted the MDMO features of the entire facial region as macro-expression features.

### Multiple linear regression analysis

Multiple linear regression analysis is a statistical method for analyzing data that aims to determine whether two or more variables are correlated with each other. Soleymani et al. (2015) analyzed the correlation between EEG signals and facial expressions using multiple linear regression methods. The purpose of using multiple linear regression analysis in this study was to explore how much of the variation in the EEG signal was correlated with facial movements when micro-expressions or macro-expressions were present. We estimated EEG features from facial features, including facial micro-expression and facial macro-expression features.

### Granger causality test

Facial EMG artifacts are a common problem in EEG recordings, in which facial muscle activity affects EEG signal recordings to some extent. In this study, we aimed to determine the extent to which muscle artifacts caused by micro-expressions or macro-expressions affect the EEG signal. To test the relationship between the two time series, we used the Granger causality test (Granger, 1969).

The Granger causality test relies on the variance of the best least-squares prediction using all information at some point in the past. Granger states that (Granger, 1969), if adding a time series *x* to an autoregressive time series *y* reduces the variance of the prediction error of *y*, then an *x* Granger causes *y* (i.e., *x* is the cause of *y*). Given a time series *y* = {*y*_1_,*y*_2_,…,*y*_*n*_}, the autoregressive model is described by Eq. 6:


(6) $$y_{i}=\sum_{j=1}^{k_{y}}\omega_{\left(k_{y}-j\right)}\,y_{\left(i-j\right)}+\epsilon_{y}.$$

where *k_y_* is the model order of the autoregressive time series, which is calculated using model criteria, such as the Akaike and Bayesian information criteria, ω_(*k*_*y*_−*j*)_ are the model coefficients, and ϵ_*y*_ is the vector of the white-noise value. The time series *x* is as shown in Eq. 7.


(7) xi=∑j=1kxω′(kx-j)⁢x(i-j)+ϵx.

Adding the lag value *x* to the regression equation for *y*, that is, reconstructing *y*, as shown in Eq. 8:


(8) yi=∑j=1kyω(ky-j)⁢y(i-j)+∑j=1kxω″(kx-j)⁢x(i-j)+ϵxy.

We determine whether Granger causality exists by using the *F*-test of the following *F*-value:


(9) $$F=\frac{\left(\epsilon_{y}^{2}-\epsilon_{x\,y}^{2}\right)/k_{x}}{\epsilon_{x\,y}^{2}/\left(n-\left(k_{x}+k_{y}+1\right)\right)}.$$

Before performing the Granger causality test analysis, we resampled the EEG signals to 1,000 Hz to match the sampling rate of the facial expressions. Thereafter, for macro-expressions, we performed Granger causality tests from the entire facial region (i.e., for *x_i_* as one dimension) to 128 channels of EEG signals (i.e., for *y_i_* as 128 dimensions). For micro-expressions, we performed Granger causality tests from each of 13 facial regions ROIs (i.e., for *x_i_* as 13 dimensions) to each of 128 EEG signals from all the electrodes separately (i.e., for *y_i_* as 128 dimensions). In this study, we used the *F*-test at an α = 5% level of significance to determine whether Granger causality exists.

## Results

### Correlation analysis

**FIGURE 4:**
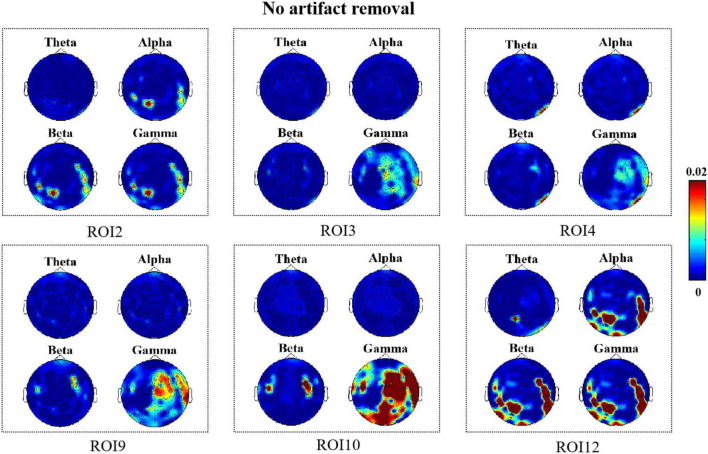
*R*^2^ topography depicts how many electroencephalography (EEG) features can be estimated as a result of facial movements (no artifact removal).

**FIGURE 5:**
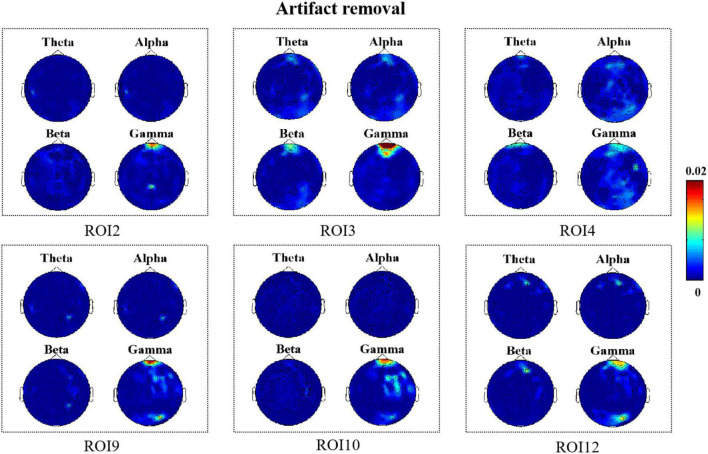
*R*^2^ topography depicts how many electroencephalography (EEG) features can be estimated as a result of facial movements (artifact removal).

In this section, we show the results of the correlation between micro-expression features for 13 different facial ROIs and EEG features for these two cases when no artifact removal was performed on the EEG signal and after artifact removal, respectively. PSD was extracted as the EEG feature from different bands and the MDMO features extracted from each frame and were averaged as facial features. The *R*^2^ results of the EEG features estimated from the micro-expression features of the 13 different facial ROIs without artifact removal are shown in Figure 4. The correlation results after artifact removal are shown in Figure 5. Here, we only show the significant *R*^2^ results when estimating EEG features from the facial features.

As shown in Figure 4, we observed significant *R*^2^ results in the facial ROI2, ROI3, ROI4, ROI9, ROI10, and ROI12 regions when no artifact removal was performed on EEG signals. This indicated that when micro-expressions occurred, changes in EEG signals were closely associated with muscle activity near the eyebrows, mouth, and chin. Notably, there were some differences in the brain regions where changes in EEG signals were correlated with the presence of facial muscle activity in these six ROIs; however, we found that high-frequency components located mainly in the frontal and temporal regions were all closely associated with facial muscle activity in these six regions. In addition, we found that for a few ROIs, namely, ROI10 and ROI12 (lips, chin), high-frequency components located in the occipital region were associated with facial muscle movements elicited when micro-expressions appeared. The high-frequency components located in the parietal region were also associated with muscle movements in ROI9 and ROI10 (mouth).

According to Figure 5, we found that when artifacts were removed from the EEG signals in all six regions, it was mainly the high-frequency components located in the frontal region that were correlated with the facial muscle movements caused by micro-expressions. Moreover, comparing Figures 4, 5, we found that there was a decrease in the degree of correlation after artifact removal as compared with when no artifact removal was performed on the EEG signals.

### Analysis of the effect of artifacts

**FIGURE 6:**
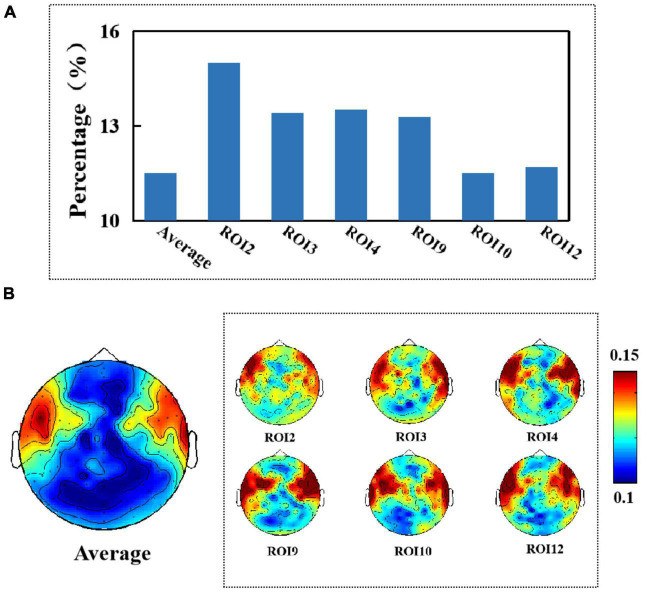
**(A)** Average percentage of significant causal relationships of facial ROIs for different electroencephalography (EEG) signals. **(B)** Topography of affected brain regions (no artifact removal).

First, we show the results of the average percentage of positive causality tests for different electrodes when testing for causality from facial micro-expressions for different EEG signals (when no artifact removal was performed on the EEG signals). We found that an average of 11.5% of the causality tests were positive when we tested whether the EEG signal was Granger-caused by facial micro-expressions. Muscle activity in the facial ROI2, ROI3, ROI4, ROI9, ROI10, and ROI12 regions (i.e., the brow, mouth, and chin regions) had the greatest effect on the EEG signal by up to 15%, as shown in Figure 6A. In addition, we found that all electrodes located in the frontal and temporal regions were significantly affected. The results are shown in Figure 6B.

**FIGURE 7:**
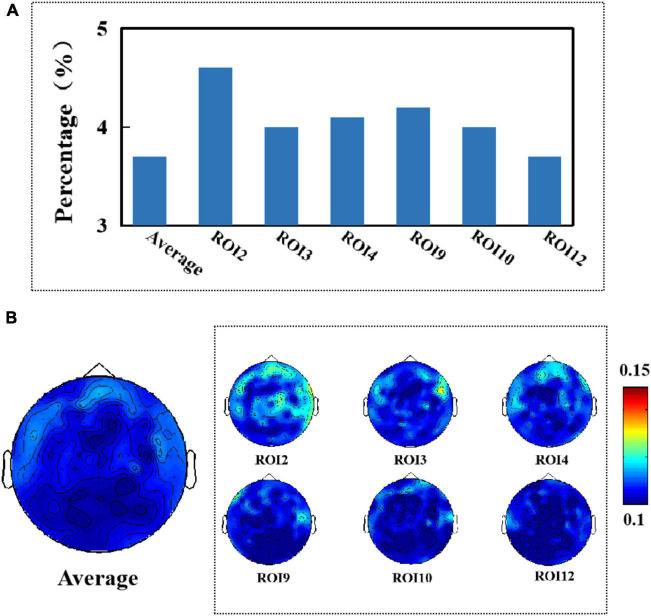
**(A)** Average percentage of significant causal relationships of facial ROIs for different electroencephalography (EEG) signals. **(B)** Topography of affected brain regions (artifact removal).

After removing artifacts from the EEG signal, we found that the average percentage of the EEG signal affected by micro-expressions from 13 different sub-regions was 3.7%, and the maximum percentage of the degree of effect was 4.6%, as shown in Figure 7A. All electrodes on the frontal region were affected, as shown in Figure 7B. Comparing Figures 6, 7, we found that compared to when the artifacts were not removed, the EEG signal was affected by muscle artifacts caused by facial micro-expressions to a lesser extent, and fewer areas were affected when the artifacts were removed.

Finally, we noted that they were similar to the results of the correlation analysis. This further reinforces the idea that a significant part of the variance in the EEG signal was caused by facial muscle artifacts. Moreover, after artifact removal from the EEG signal, the EEG signal was much less affected by muscle artifacts.

## Discussion

In this study, we examined the effect of facial muscle artifacts caused by micro-expressions on EEG signals. First, we recorded facial expressions and EEG signals of 68 college participants. We subsequently extracted the MDMO features of facial micro-expression sequences and EEG features separately. Additionally, we investigated the correlation between EEG features and facial MDMO features to find possible cross-model effects of facial muscle activities. Finally, Granger causality statistical analysis was performed to determine the extent to which the EEG signal was affected by facial muscle artifacts (induced by facial micro-expressions) when micro-expressions were present. The results showed that the average percentage of EEG signals affected by muscle artifacts caused by facial micro-expressions was 11.5% (when no artifacts were removed from the EEG signal). Specifically, the temporal and frontal regions were significantly affected by muscle artifacts when compared to other brain regions. Compared to other facial regions, the muscle activity in the mouth and eyebrow regions had the strongest effect on EEG signals. When artifacts were removed from the EEG signals, the average percentage of EEG signals affected by muscle artifacts caused by facial micro-expressions was 3.7%, with the frontal region being the most affected.

Notably, the results of the present study correlate with those of Goncharova et al. (2003) and Liong et al. (2015). Liong et al. (2015) showed that the eyebrows and the mouth were the most effective facial regions when micro-expressions occurred. Goncharova et al. (2003) showed that frontal muscle contraction significantly affected frontal regions and that temporal muscle contraction significantly affected temporal regions. In our study, we also found that by observing the facial responses of the participants, there were changes in muscle activity near the eyebrows and mouth (in most participants) when micro-expressions were present, which affects the EEG signal. Thus, this again confirms our results. That is, the frontal and temporal regions were significantly affected compared to other brain regions. Muscle activity in the mouth and eyebrow regions had a greater effect on the EEG than in other facial regions. Furthermore, by removing artifacts from the EEG, it was found that the degree of this effect decreased to 3.7%. The comparison of the two effect results revealed that the EEG signal was less affected by facial muscle artifacts after artifact removal.

**FIGURE 8:**
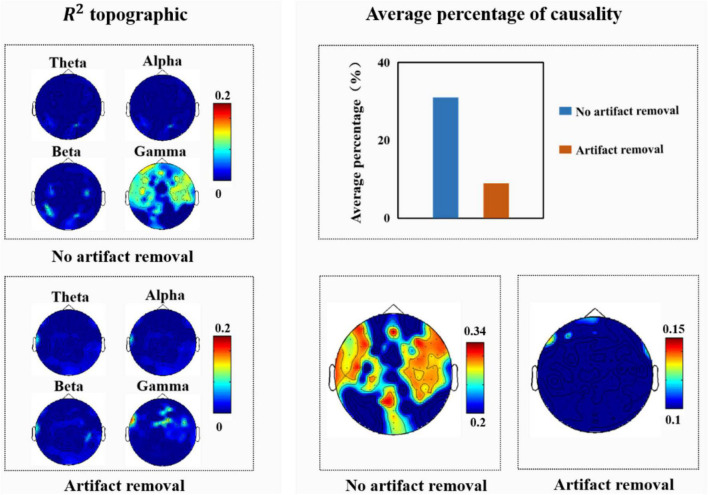
**(Left)** *R*^2^ topography of electroencephalography (EEG) features estimated from facial features. **(Right)** Average percentage of significant causal relationships of facial ROIs for different EEG signals.

Since Soleymani et al. (2015) analyzed the effect of muscle artifacts induced by macro-expressions on EEG signals, they concluded that the average percentage of facial muscle artifacts caused by macro-expressions on EEG signals was 54%, with the frontal, parietal, and occipital regions being significantly affected. Therefore, in this study, we also analyzed the experimentally collected macro-expressions and EEG signals; the results of the correlation and causality analyses are shown in Figure 8. As shown Figure 8, we observed that high-frequency components located mainly in the frontal, temporal, and occipital regions were closely related to the facial muscle activity elicited when macro-expressions appeared. After further causality test analysis, it was found that an average of 31% of the causality tests were positive when testing whether the EEG signal was Granger-induced by facial macro-expressions, with the frontal, temporal, and occipital regions being significantly affected. There were some differences between these results and the results of the Soleymani et al. (2015).

We believe that the possible reasons for these differences are: (1) the purpose of the experimental design was different. Our experiment intended to induce spontaneous micro-expressions in the participants, who were requested to try their best to suppress their real emotions during the experiment. Therefore, the intensity of macro-expressions in this study was lower than normal macro-expressions. (2) This study only analyzed the facial responses produced by participants while watching videos with positive emotions, whereas Soleymani et al. (2015) analyzed the facial activities produced by participants while watching videos with different emotions (positive, negative, etc.). In addition, we further analyzed the EEG signals after removing artifacts and found that high-frequency components, mainly located in the temporal region, were closely associated with facial muscle activity elicited when macro-expressions were present. The average percentage of EEG signals affected by macro-expressions was 9%, and the frontal region was affected. This indicated that the EEG signal was less affected by artifacts when they were removed.

Our findings provide a basis for emotion recognition based on EEG and micro-expressions. In recent years, researchers have started to use EEG signals and micro-expressions to recognize emotion, but the degree and scale of contamination by artifacts on the EEG signal caused by micro-expressions remains unclear. In our study, the experimental results showed that the EEG signal was less affected by facial muscle artifacts after artifact removal than when artifacts were not removed. This suggested that when artifacts were removed from the EEG signal, the EEG signal used was mainly generated by neural activity in the brain and there were limited effects caused by facial muscle artifacts. The results of this study demonstrate the reliability of research on emotion recognition based on EEG signals and micro-expressions, which provide novel insight into emotion recognition based on EEG signals and micro-expressions.

However, our study has some limitations. First, our participants were all college students from China, hence, we did not consider any differences between ethnic groups and ages. Future studies should consider inviting participants from multiple ethnic groups and multiple ages to participate in the experiment to ensure diversity across ethnic groups and ages. Second, our study only considered one emotion label, namely, positive emotion, and did not investigate other emotions. This is because Johnston et al. (2010) argued that smiling required little preparation and smiling was usually used more frequently than other emotional expressions. Therefore, future studies can attempt to induce many different emotions for analysis, such as anger and sadness. Finally, to the best of our knowledge, the present study was the first study to explore the effect of micro-expression-induced artifacts on EEG signals, and future studies could further validate our results.

## Conclusion

In this study, we investigated whether an EEG signal is affected by facial artifacts caused by micro-expressions by analyzing the relationship between micro-expressions and the EEG signal. The results showed that the average percentage of EEG signals affected by facial artifacts caused by micro-expressions (when the artifacts were not removed) was 11.5%, and that the frontal and temporal regions were significantly affected. The average percentage of EEG signals affected after artifact removal was 3.7%, and the frontal regions were affected. The EEG signal was less affected by facial muscle artifacts after artifact removal than when the artifacts were not removed. The experimental results demonstrated that the EEG signals produced by micro-expressions were mainly generated by brain neural activity and there were limited effects caused by facial muscles artifacts. This study lays the foundation for emotion recognition based on micro-expressions and EEG signals.

## Data availability statement

The original contributions presented in this study are included in the article/supplementary material, further inquiries can be directed to the corresponding author.

## Ethics statement

The studies involving human participants were reviewed and approved by Ethics Committee of Southwest University. The patients/participants provided their written informed consent to participate in this study. Written informed consent was obtained from the individual(s) for the publication of any potentially identifiable images or data included in this article.

## Author contributions

All authors listed have made a substantial, direct, and intellectual contribution to the work, and approved it for publication.
